# Inflammatory pseudotumor secondary to urachal cyst: A challenging clinical case report^⋆^

**DOI:** 10.1016/j.ijscr.2019.12.029

**Published:** 2019-12-26

**Authors:** Valerie Armstrong, Kristina Khazeni, Andrew Rosenberg, Sanjaya K. Swain, Mecker Moller

**Affiliations:** a University of Miami Miller School of Medicine, Miami, FL, United States; b University of Miami General Surgery Department of Surgical Oncology, United States

## Abstract

**Introduction:**

There is a wide differential diagnosis for intraabdominal tumors. Surgical resection and microscopic analysis of tissue structure can identify tumor origin in most cases. Most rapidly growing invasive tumors are neoplastic. Inflammatory pseudotumors are a subcategory of intraabdominal tumors that are non-neoplastic and can be rapidly growing. Urachal cysts originate from the dome of the bladder; however they are typically not invasive. There is limited literature on the appropriate management of these tumors.

**Presentation:**

A 37-year-old female presenting with symptoms of abdominal pain was found to have a large intraabdominal mass invading multiple organs. Core biopsies demonstrated inflammation. The mass grew significantly over the course of a year and patient's abdominal pain worsened. The patient was taken to the operating room for resection. Final pathology revealed reactive fibrous tissue with acute and chronic inflammation invading bladder, urethra, abdominal wall, appendix, and ovary. Intraoperative frozen section demonstrated low grade spindle cells with concern for inflammatory pseudotumor but final pathology demonstrated inflammation.

**Discussion:**

Although benign, these tumors cause significant morbidity due to their size and level of organ invasion. Management should involve surgical resection as well as potential post-operative chemotherapy or NSAIDs based off clinical picture. We demonstrate the importance of close follow up for residual disease or recurrence of patients with inflammatory pseudotumors of the abdomen.

**Conclusion:**

This case highlights difficulties in diagnosis of a tumor that has potential to cause significant morbidity. There is need for further research to discover the best management after surgical resection of these tumors.

## Highlights

• Inflammatory pseudotumor are non-neoplastic tumors of the abdomen.
• They may be large and invasive.
• They may cause significant morbidity and mortality.
• There is limited information in the literature about management.

## 1 Introduction

Mesenchymal tumors of the pelvis and abdomen represent a heterogeneous group of neoplastic and non-neoplastic lesions. Differentiating among these different lesions is clinically important due to the wide range of prognosis and treatment. The rare group of tumors composed of fibroblastic or myofibroblastic cells represent reactive pseudoneoplastic tumors and may be benign or malignant [1]. Case reports describe few cases of mesenchymal tumors arising from the urachus [2]. We present a case of a large intraabdominal mass in a 37-year-old female involving the abdominal wall, bladder, ovary, and ureter; pathologically identified as reactive fibrous tissue with acute and chronic inflammation. The details of the patient’s clinical presentation, diagnostic steps, radiology, analysis of histology, operative and postoperative management were reviewed.

Urachal remnants that are asymptomatic are observed and managed conservatively. Those that are symptomatic or become infected are traditionally excised surgically [3]. It is important to evaluate all urachal remnants for potential malignancies such as urachal carcinoma or bladder cancers including urothelial carcinoma, squamous cell carcinoma, or adenocarcinoma, as malignant pathology changes prognosis and management [4]. The work done for this case report is in line with SCARE criteria [12].

## 2 Case presentation

**Fig. 1:**
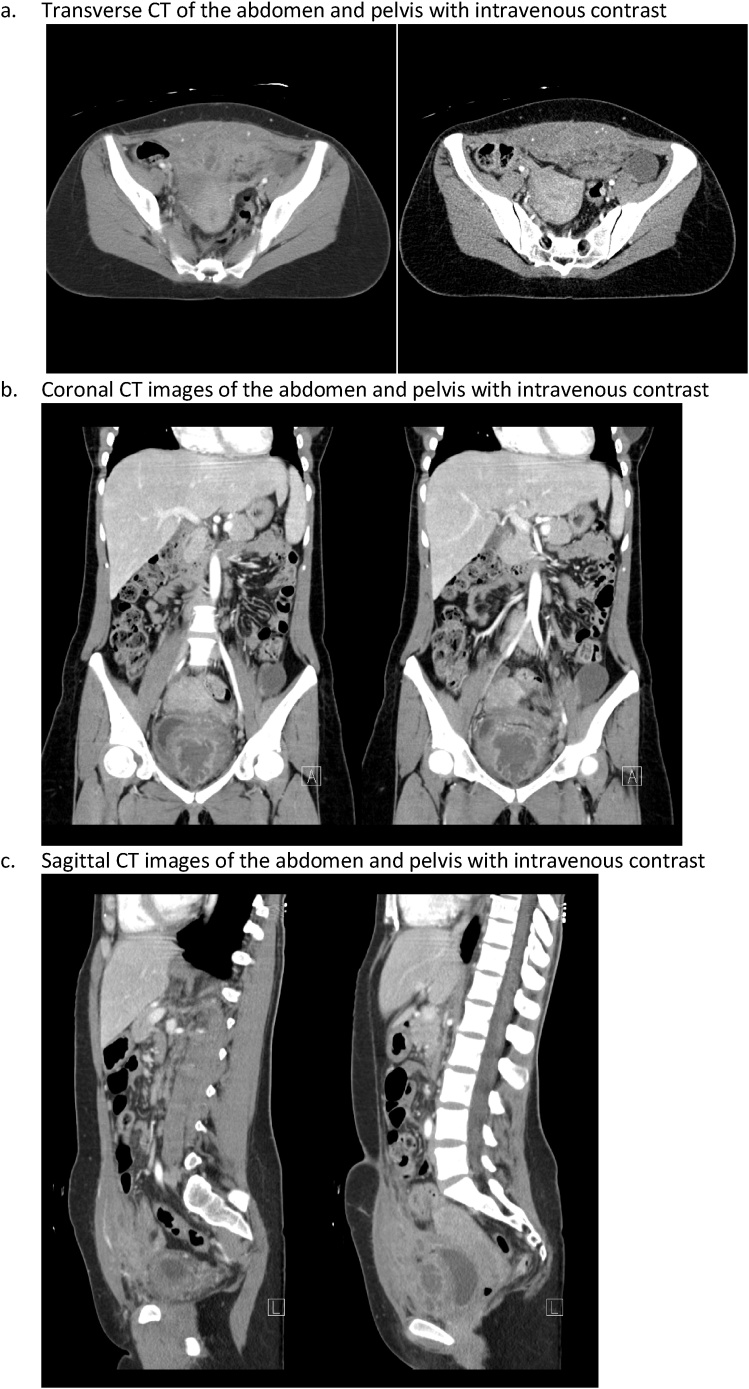
Radiographic images.

A 37-year-old female presented to the emergency room at our institution with intractable abdominal pain and fever. She has a history of dysuria and hematuria for 10 months, which at the time prompted an MRI demonstrating an abdominal wall mass with extension into the bladder. Outside biopsy was reported as “inflammation”. She was initially treated with antibiotics. A cystoscopy was performed and a transurethral bladder biopsy showed acute and chronic cystitis and Von Brunn nests. Cytology report indicated presence of atypical urothelial cells and she was then referred to the urology service due to suspicion for malignant neoplasm. Follow up CT scan imaging demonstrated a heterogeneous ill-defined enhancing soft tissue density in the space of Retzius inseparable and compressing the anterior aspect of the urinary bladder measuring 11.3 × 4.5 × 12.8 cm with a peripherally enhancing loculated collection measuring 4.7 × 6.0 × 3.0 cm along with marked thickening of urinary bladder Fig. 1. As strong suspicion for malignant lesion persisted, a repeat percutaneous biopsy was done that again demonstrated infiltrative granulation tissue and chronic inflammation.

As the patient’s symptoms continued to progress, including persistent hematuria diagnosis was uncertain despite multiple percutaneous and transurethral biopsies, she was referred to Surgical Oncology team for multidisciplinary management. As there were still concerns for neoplastic malignant process vs desmoid vs pseudoinflammatory neoplasm, the multidisciplinary team recommendations were to offer operative management in coordination with the surgical oncology and urology teams with an intention to provide larger tissue for definitive diagnosis to guide extend of surgery, which would include complete bladder removal with construction in the form of ileal conduit, abdominal wall resection with abdominal wall reconstruction if malignancy were to be confirmed intraoperatively.

### 2.1 Surgery

**Fig. 2:**
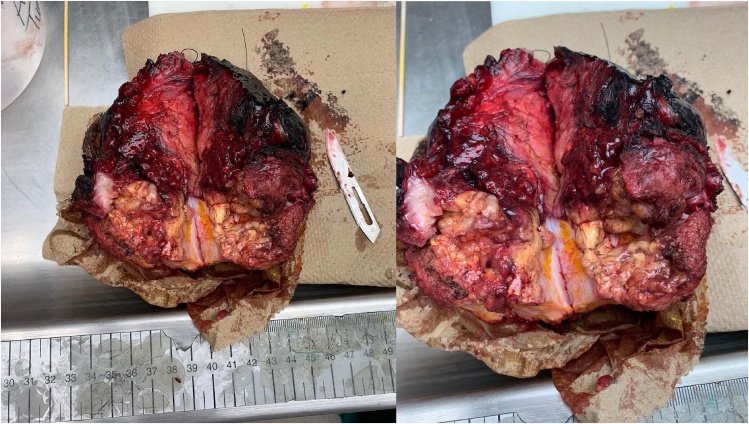
Gross pathologic images: intraoperative imaging of excised tumor. Well circumscribed heterogeneous tumor invading multiple organs, measuring 11.3 × 4.5 × 12.8 cm.

Intraoperative cystoscopy demonstrated an ulcerated infiltrative tumor into the dome of the bladder. Intraoperative frozen biopsies did not provide definite diagnosis and suggested spindle cell neoplasm, possible of urachal origin but malignancy could not be confirmed, thus the decision was to proceed with palliative resection including abdominal wall resection with en bloc resection of pelvis tumor, partial cystectomy and partial oophorectomy, as the mass was found to have invaded the abdominal wall below the umbilicus as well as the ipsilateral ovary and ureter. There was significant thickened white tissue within the muscles and extending to posterior rectus sheath. Fig. 2. However, near the bladder the abnormal thickened tissue extended from the dome of the bladder over the urethra. Decision was made to avoid creation of an ileal conduit and rather perform a partial cystectomy and await definitive diagnosis. An incidental Meckel’s diverticulum was found and resected.

### 2.2 Pathology

The final pathology showed an ill-defined pale-tan markedly fibrotic area measuring 8.5 × 5 × 5 cm extending from the mucosa. The mucosal and submucosal aspect of the bladder showed markedly necrotic aspects. The abdominal rectus muscles were grossly present in the specimen and were focally involved by the fibrotic area. The abdominal wall mass was predominantly reactive fibrous tissue with acute and chronic inflammation and fibrosis without evidence of malignant neoplasia. The mass contained low-grade spindle cell proliferation with abundant inflammation, granulation tissue, and chronic abscess. The mass had connections to the bladder suggesting urachal origin. The muscularis layer was noted to have attached granulation tissue. Left partial oophorectomy yielded unremarkable parenchyma with attached reactive fibrous tissue containing chronic inflammation and hemorrhage.

**Fig. 3:**
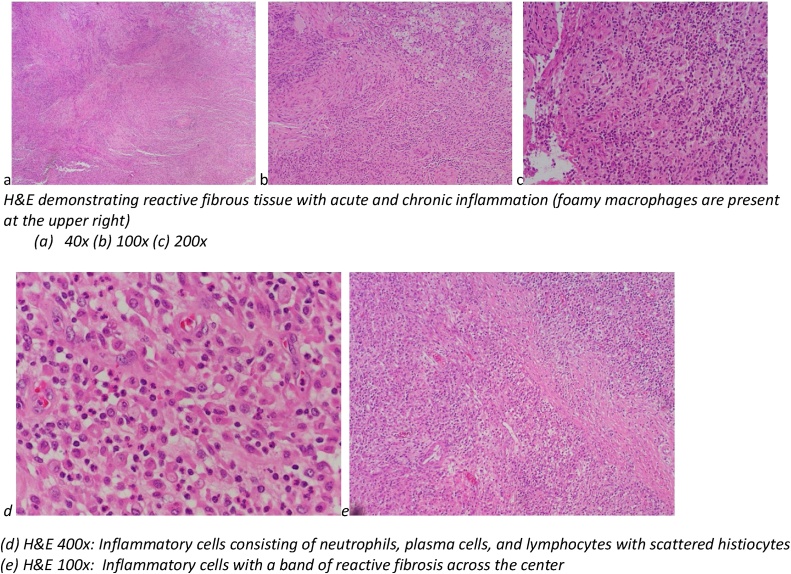
Microscopic pathologic images.

The frozen section demonstrated an ill-defined pale-tan markedly fibrotic area measuring 8.5 × 5 × 5 cm extending from the bladder mucosa. The mucosal and submucosa of the bladder showed markedly necrotic aspects. The abdominal rectum muscles were grossly present and were focally involved by the fibrotic area (Fig. 3).

### 2.3 Clinical outcome

The patient’s final pathology was discussed in multidisciplinary tumor board and recommendations were to treat her with NSAIDS to attempt control residual disease if symptoms returns or progression occurs. A post-op MRI 10 months later showed resolving residual inflammation. She is recommended to remain on NSAIDs and close follow up and monitoring her with imaging.

## 3 Discussion

Tumors of the abdominal cavity can be particularly difficult to diagnose and manage. In the case described here, the top differential diagnoses included urachal cyst and inflammatory pseudotumor, however this tumor was unique in its invasive properties and rapid growth. Little has been documented in the literature regarding similar tumors and their management.

Tumors of similar pathology have been documented and include reactive nodular fibrous pseudotumors of the gastrointestinal tract, inflammatory myofibroblastic tumors, retroperitoneal fibrosis, sclerosing mesenteritis [5,6], nodular fasciitis, transabdominal fibromatosis, intraabdominal inflammatory myofibroblastic tumor, inflammatory fibrosarcoma [[7], [8], [9]], and GIST. These tumors, however, rarely involve multiple organs.

Inflammatory myofibroblastic tumors have been documented to be invasive in cases. These tumors have the highest incidence in children and adolescents. They are classified as benign; however, they have been documented to invade multiple organ systems and metastasize. Surgical resection, when feasible, is the recommended management. Unresectable tumors may be sensitive to chemotherapy and have been documented to spontaneously regress in some cases [10]. Success has also been shown with use of NSAIDs for treatment [11].

The urachus is a structure that normally involutes at 6 months gestation and becomes the umbilical ligament. Urachal remnants typically present in adulthood with infection with or without abscess formation, or rarely with formation of malignant tumors. Microscopically, these tumors appear similar to the tumor described in our case report – with inflammatory tissue and spindle cells in collagenous stroma. A urachal remnant *should* be discernable within the mass as an area of glandular tissue [2].

Immunohistochemical staining can play a diagnostic role in tumors of unknown origin, and is particularly useful when the final pathology is indicative of neoplasm. Reactive nodular fibrous pseudotumors of the gastrointestinal tract are commonly known to stain positive for CD117, which is also seen in many myofibroblastic proliferations and gastrointestinal stromal tumors. Other commonly positive stains in pseudotumors of the gastrointestinal tract include vimentin, muscle specific actin, smooth muscle actin, desmin, S-100 protein, CD34, ALK-1, and keratin [1].

This case report highlights the importance of determining appropriate management of non-neoplastic, invasive, inflammatory abdominal tumors of unknown origin. Decisions to biopsy based on initial imaging, as well as decisions to operate based on pathology should be further evaluated.

## 4 Conclusion

Prognosis of urachal cyst versus malignant inflammatory pseudotumor varies greatly; thus, proper diagnosis is of critical importance. Initial management, treatment, and long term follow up for tumors composed of reactive fibrous tissue is not well documented in the literature. Our patient represents a difficult case in management both preoperatively and postoperatively due to vague clinical presentation and inconclusive biopsies suggesting an infiltrative neoplasm. Close follow up imaging and clinical monitoring are recommended to determine future progression of disease and response to therapy. In patients with recurrence of inflammation from urachal cyst or residual disease, there is a need for further management other than surgery. These patients benefit from multidisciplinary discussion and management.

## Sources of funding

NA.

## Ethical approval

Projects that do not meet the federal definition of research pursuant to 45 CFR 46 do not require IRB review. This tool was developed to assist the University of Miami community in determining when a project falls outside of the IRB’s purview.

## Consent

Written informed consent was obtained from the patient for publication of this case report and accompanying images. A copy of the written consent is available for review by the Editor-in-Chief of this journal on request.

## Author contribution

VA – literature search, case report design, gathering history, data interpretation.

KK – literature search, gathering history, data interpretation, editing.

AR – pathology report, images.

SS – review and additions to manuscript.

MM – literature search, case report design, gathering history, data interpretation.

## Registration of research studies

Not a clinical trial.

## Guarantor

Dr. Mecker Moller.

## Search terms

Pseudotumor, invasive, intraabdominal, inflammatory, urachal cyst, invasion, invasive, invading multiple organs, extension, contiguous spread, contiguous extension.

## Provenance and peer review

Not commissioned, externally peer-reviewed.

## Declaration of Competing Interest

N/A.
